# Hypoxia-mediated activation of hypoxia-inducible factor-1α in triple-negative breast cancer: A review

**DOI:** 10.1097/MD.0000000000035493

**Published:** 2023-10-27

**Authors:** Lihui Liu, Jie Bai, Lanxin Hu, Daqing Jiang

**Affiliations:** a Liaoning University of Traditional Chinese Medicine, Shenyang, China; b Department of Breast Surgery, Cancer Hospital of Dalian University of Technology, Cancer Hospital of China Medical University, Liaoning Cancer Hospital and Institute, Shenyang, China; c The Affiliated Traditional Chinese Medicine Hospital of Southwest Medical University, Luzhou, China.

**Keywords:** HIF-1, HIF-1α, hypoxia, signaling pathways, TNBC

## Abstract

Triple-negative breast cancer (TNBC) is a subtype of breast cancer (BC) that is highly aggressive and hypoxic compared with other subtypes. The role of hypoxia-inducible factor 1α (HIF-1α) as a key hypoxic transcription factor in oncogenic processes has been extensively studied. Recently, it has been shown that HIF-1α regulates the complex biological processes of TNBC, such as glycolysis, angiogenesis, invasion and metastasis, BC stem cells enrichment, and immune escape, to promote TNBC survival and development through the activation of downstream target genes. This article discusses the expression of the HIF-1α transcription factor in TNBC and the Hypoxia-mediated activation of hypoxia-inducible factor-1α in triple-negative BC. It offers a fresh approach to clinical research and treatment for TNBC.

## 1. Introduction

Breast cancer (BC) is the most common female malignancy.^[1]^ Triple-negative breast cancer (TNBC) is a subtype of breast tumor lacking hormone receptors expression and human epidermal growth factor receptor-2 gene amplification and accounts for approximately 15% to 20% of all breast carcinomas.^[2]^ TNBC is extremely hostile, and about 46 percent of patients get remote metastases, which typically affect the internal organs and brain.^[3–5]^ In addition, TNBC has a 40 % mortality rate within 5 years of diagnosis and a high probability of recurrence.^[6]^ Just 13.3 months on average are spent alive following metastasis, and up to 25% of patients experience recurrence after surgery.^[7]^ TNBC, however, is unable to respond to endocrine or single - molecule therapy because of its specific molecular subtype. Conventional chemotherapy, which is currently the primary treatment for TNBC, is susceptible to drug resistance and provides only marginal therapeutic benefits.^[8]^ Therefore, finding therapeutic targets for TNBC is an urgent clinical problem to be solved.

Radiation and chemo-resistance are frequently caused by hypoxia, which is a common occurrence in malignancies. It is well known that hypoxia prevents differentiation and promotes invasion and metastasis. Research has demonstrated that intra-tumoral hypoxia is linked to more aggressive phenotypes, a greater likelihood of metastasis, chemotherapy, and immunotherapy resistance, and has a detrimental effect on BC patients long-term survival.^[9]^ Importantly, TNBC has a more prominent hypoxic signature than other BC subtypes.^[10,11]^ Inflammatory mediators generated by TNBC, the hypoxic microenvironment, numerous signaling pathways, and other variables all have a significant role in the regulation of HIF-1, a critical transcription factor for hypoxia, and these interactions also demonstrate the complicated biological behavior of hypoxia-inducible factor 1α (HIF-1α).^[12]^ By focusing on downstream genes, HIF-1α governs the intricate biochemical processes of TNBC, slowing down its development.

## 2. HIF-1α expression and research status in the TNBC

Recent studies have shown that the HIF-1 pathway is overactive in TNBC, compared to other isotypes, and that the HIF-1 protein is overexposed in more than 80% of TNBC patients.^[13,14]^ Several signaling pathways, noncoding RNA, inflammatory mediators secreted by tumor cells, in the TNBC tumor microenvironment of oxygen levels, and other factors could all play a role. Moreover, epigenetic changes control HIF-1’s expression. Several factors control HIF-1’s expression, which is more favorable in TNBC. For instance, HIF-1 is expressed on the surface of cancer cells through its interaction with chemokines, and also can be activated by cytokine receptors. Without a sufficiently high drug concentration locally, even a normoxic tumor microenvironment does not guarantee that all tumor cells are sensitive to chemotherapy, especially when treated tumor cells activate survival molecules such as HIF-1. To determine the effects of doxorubicin on normoxic HIF-1a accumulation, we selected several doxorubicin concentrations in the ranges of in vivo drug concentrations in 4T1 and MCF-7 tumors.

### 2.1. Structure of HIF-1

**Figure 1.**
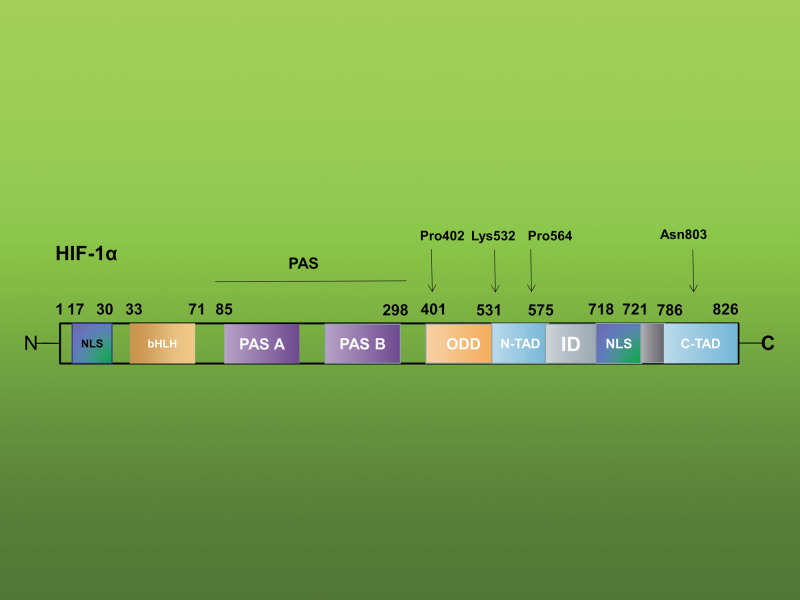
Schematic representation of structure of HIF-1a. The figure has been adapted, with some modifications, from Li and Ye.^[15]^ Numbers indicate the amino-acid residues of each domain or special amino sites. From N-terminal, HIF-1a contains nuclear localization signals (NLS), a basic helix-loop-helix (bHLH) domain and two Per-ARNT-Sim homology domains (PAS-A and PAS-B). N- and C-terminal transactivation domains (N-TAD and C-TAD) are separated by an inhibitory domain (ID).

HIF-1 (Fig. 1) is a heterodimer made up of 2 subunits: HIF-1α, which is expressed constitutively, and HIF-1b is a member of the protein family known as basic helix-loop-helix-Per-ARNT-Sim.^[16]^ HIF-1α is composed of the oxygen-dependent degradation domain (ODDD), which confers oxygen-dependent regulation, the bHLH and PAS domains (PAS-A and PAS-B), which are necessary for dimerization and DNA binding, as well as 2 independent transcriptional activation domains, the N- and C-terminal transactivation domains (N-TAD and C-TAD), which are separated by an inhibitory domain.^[17]^ nuclear localization signals -N(N-NLS) and nuclear localization signals -C (CNLS), 2 nuclear localization signals, are also present in HIF-1α. Whereas the N-terminal NLS appears to be less significant, the C-terminal NLS is essential for the nuclear import of HIF-1α. Due to its structural similarity, the HIF-1b component plays a crucial role in a number of transcriptional processes and is essential for HIF-1 DNA binding and transactivation.^[18]^

### 2.2. HIF-1α regulates the progression of TNBC

The intricate process through which HIF-1 regulates TNBC encompasses numerous signaling channels and associated processes. In this case, HIF-1 is primarily regulated by the introduction of inflammatory mediators and oxygen levels, as this is a very important and proactive strategy to manage HIF-1. And also, HIF-1α resulted in the biological processes of glycolysis, angiogenesis, invasion, and metastasis, tumor stem cell enrichment and immunological escape.

#### 2.2.1. Inflammatory mediators and oxygen levels to regulate HIF-1α.

HIF-1α activity can be regulated by proinflammatory mediators produced by TNBC cells and the hypoxic tumor microenvironment. Inflammatory cells can release chemicals, especially reactive oxygen species, which can actively mutate surrounding cancer cells and accelerate their transformation into highly malignant tumors. TNBC is a very hypoxic solid tumor.^[19]^ The development of the hypoxic microenvironment inside the tumor is accelerated by the increased oxygen consumption of the tumor cells and the insufficient oxygen delivery provided by the tumor microvasculature.^[20]^ TNBC cells produce reactive oxygen species (ROS) and nitric oxide (NO) in response to acute and ongoing inflammation as well as the upkeep of chronic hypoxia.^[21]^

Inflammatory mediators are produced as a result of tumor-intrinsic gene mutations, inactivation, and antitumor therapy for TNBC. In clinical practice, commonly used chemotherapeutic medicine for TNBC of treatment, such as cyclic medication, alkylating agents, platinum, irradiation, and other treatment approaches, could cause a considerable quantity of ROS and NO generation, creating a hypoxic microenvironment through an initial inflammatory response in turn regulates the activity of HIF-1.^[22,23]^ Additionally, TNBC-specific gene mutations and inactivations result in significant quantities of ROS production, for example, BRCA1 inactivation, TP53 alterations, and stem cell-like characteristics.^[24]^ Particularly, TP53 mutations cause elevated NO levels. Under typical circumstances, P53 builds up, triggers apoptosis in response to NO-mediated DNA damage, and inhibits iNOS via a regulatory route of negative feedback. The negative feedback regulatory system is broken when TP53 is altered, which causes iNOS to be stimulated to create more NO.^[25,26]^

**Figure 2.**
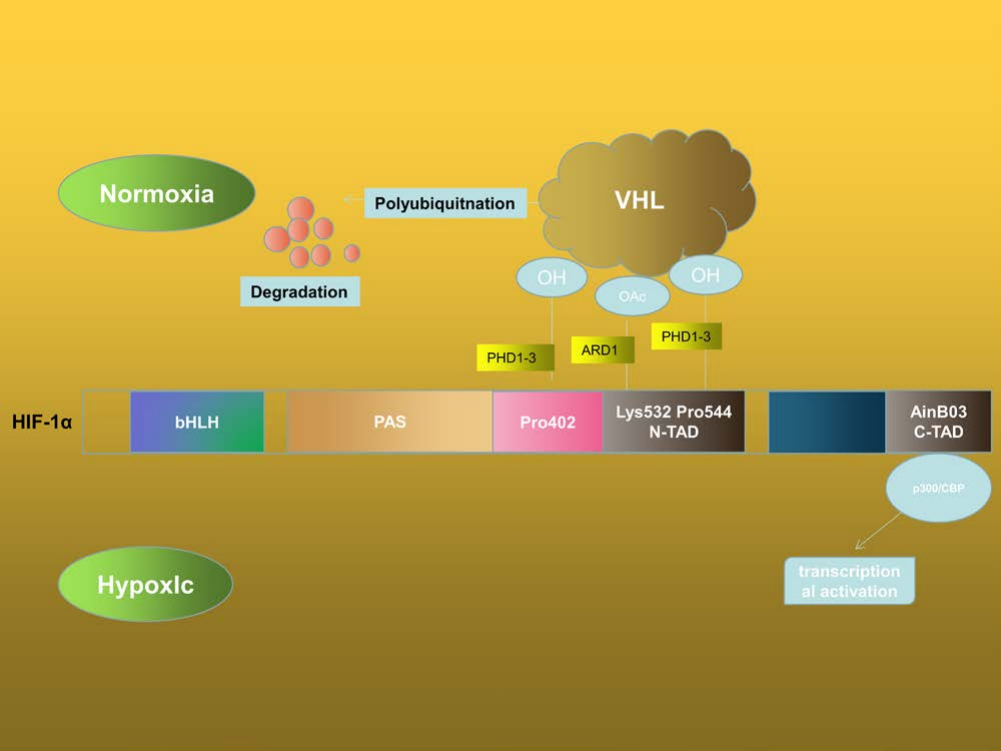
Regulation of HIF-1 at various oxygen concentrations and its domains. HIF-1 builds up in the cells as a result of the hydroxylation activity of PHD-2 failing at low oxygen concentrations (21% O_2_). A transcription initiation complex, which also detects flaws in the target gene promoters, is formed when accumulated HIF-1 translocates to the nucleus, forms a dimer with HIF-1, and binds with the transcriptional coactivator p300/CBP. Transcription is induced by the hypoxia response element (HRE). PHD-2 = hydroxylase-2.

The TNBC’s hypoxic microenvironment preserves HIF-1’s stability and activity. Prolyl hydroxylase-2 (PHD-2) hydroxylates proline residues on the N-TAD of HIF-1 in normoxic circumstances, causing HIF-1α to interact with the von Hippel-Lindau tumor suppressor protein. Ubiquitination ligase then binds to HIF-1, which is then ubiquitinated and destroyed by 26 seconds protease.^[27]^ Additionally, factor block HIF regulates HIF-1’s factor inhibit (FIH) transcriptional activity. HIF-1’s asparagine residue is inhibited by FIH, which also blocks the transcriptional coactivator p300/CBP from binding to HIF-1.^[28]^ When the oxygen concentration is <21%, PHD-2’s hydroxylation function malfunctions, which causes HIF-1α to build up in the cells.^[29]^ A transcription initiation complex, which also detects flaws in the target gene promoters, is formed when accumulated HIF-1α shifts to the nucleus, forms a dimer with HIF-1 and binds with the transcriptional coactivator p300/CBP. Transcription is induced by the hypoxia response element.^[30]^ It is important to note that FIH continues to perform its transcriptional role by suppressing HIF-1α even in the hypoxic condition (1% O_2_).^[31]^ Additionally, under chronic hypoxia, low ROS levels control HIF-1α activity. Recent research has revealed that ROS in the cytoplasm mostly limits PHD-2 and FIH activity while promoting HIF-1α stabilization^[31]^ (Fig. 2)

Unsurprisingly, a recent study also found that the protein level of HIF-1 in TNBC increased under normoxic conditions.^[32]^ NO plays an important role in controlling inflammatory mediators by maintaining the stability of HIF-1 and increasing the protein level of HIF-1. The potential mechanism of PHD-2 involves the binding of NO to the iron atom in the catalytic core, which prevents PHD-2 from being active.^[33]^ In addition, NO causes S-nitrosylation of the Cys533 location of the HIF-1 ODDD domain, which stops HIF-1 from degrading.^[34]^ Moreover, NO blocks FIH enzyme activity prevents FIH from hydroxylating HIF-1’s asparagine and stimulates the gene transcription that is downstream of HIF-1.^[35]^

### 2.3. Signaling pathway that regulates HIF-1α

**Figure 3.**
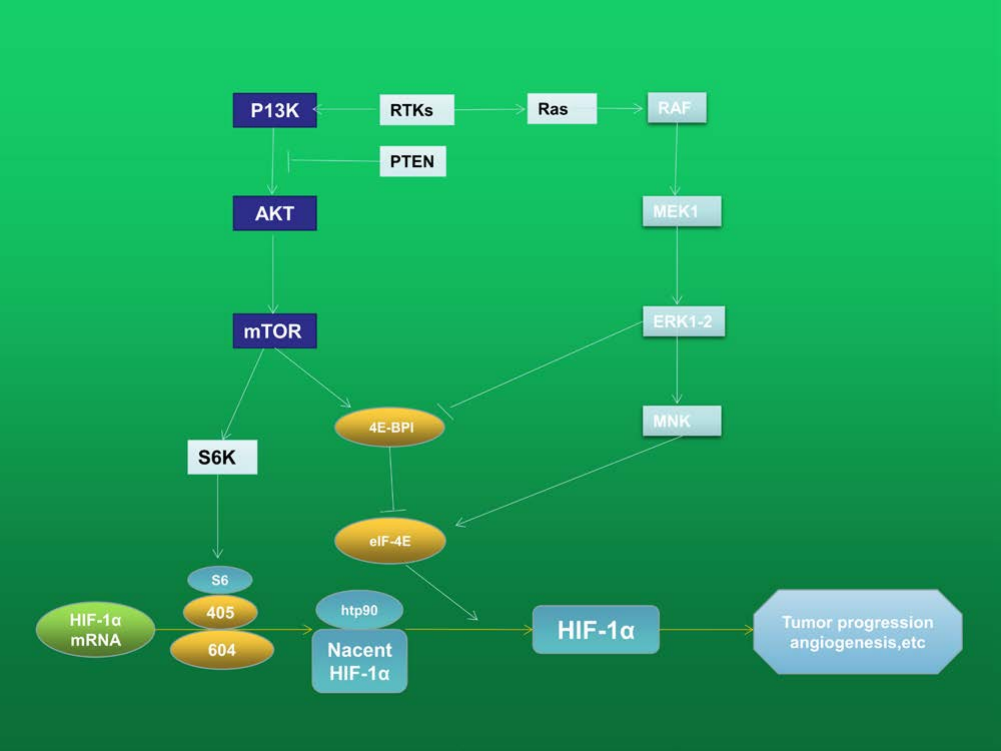
Important regulatory routes for HIF-1a translation and hypothesized HIF-1 inhibitor modes of action. The upstream MAP/ERK kinase (MEK) in the MAPK pathway activates the extracellular signal-regulated kinase (ERK). Then ERK turns on MNK. p70 S6 kinase (S6K) and the eukaryotic translation initiation factor 4E (eIF-4E) binding protein (4E-BP1) are phosphorylated by ERK and mTOR. The inactivation of eIF-4E caused by 4E-BP1 binding prevents cap-dependent mRNA translation. 4E-BP1’s ability to bind to eIF-4E is prevented by phosphorylation. MNK directly increases eIF-4E’s activity by phosphorylating it. HIF-1a-specific VHH nanobodies inhibit HIF-1a and HIF-1b binding to prevent the formation of HIF-1, which inactivates transcriptional activity.

HIF-1α, a crucial target in the control of TNBC, generates the complicated biological activity of TNBC by regulating the transcription of downstream target genes through complex signal transduction. Recent research has demonstrated that HIF-1α expression is mainly regulated by the RAS-RAF-MEK-ERK, PI3K/Akt/mTOR, and JAK-STAT signaling pathways, which in turn promote breast cancer stem cells enrichment, angiogenesis, and TNBC cell proliferation^[36–39]^ (Fig. 3). Moreover, inflammatory mediators have an impact on a portion of the signaling pathway, which further controls the production of HIF-1α. The literature points out, the PI3K/Akt/mTOR signaling pathways, RAS-RAF-MEK-ERK signaling pathways, and NF-B signaling pathways are all controlled by ROS in TNBC.^[37,40,41]^

#### 2.3.1. RAS-RAF-MEK-ERK signaling pathway.

The majority of TNBC have activated RAS-RAF-MEK-ERK, which prolongs tumor cell viability by regulating downstream caspase-mediated apoptosis pathways and is correlated with TNBC chemo-resistance and progression.^[42]^ ROS can activate ERK, because low concentrations of ROS can promote the activation of ERK through the classic Ras-Raf-MEK pathway, thereby promoting the transcriptional activation of HIF-1α. HIF-1α/p300 complex is increased by ERK phosphorylating CBP/p300, which promotes the activation of HIF-1α in TNBC.^[43,44]^ In addition, other studies have shown that ROS increases the content of HIF-1α protein by activating the ERK pathway, which is a key factor in initiating M2 macrophage polarization.^[37,41]^

#### 2.3.2. PI3K/Akt/mTOR signaling pathway.

The PI3K/ Akt/ mTOR signaling pathway is expressed in a variety of tumors. The PI3K/Akt/mTOR signaling pathway can be particularly activated by ROS, which also increases the production of HIF-1α. Translation can start when the phosphorylation levels of the translation regulators 4E-BP1 and p70S6k are changed by an active PI3K/Akt/mTOR pathway pair. When 4E-BP1 is phosphorylated, it can separate from itself and release eIF4e, which then binds to the 5’-UTR of the HIF-1α mRNA and starts translation. By encouraging the phosphorylation of the 40S ribosomal protein S6 and encouraging HIF-1α protein production, phosphorylation of P70SK6 can start translation.^[45,46]^ In some TNBC cells, Professor Tao found that anti-glycolytic compound 2-deoxy-D-glucose works in conjunction with Polydatin to suppress the ROS/PI3K/AKT/HIF-1α/HK2 signaling axis, thus inhibiting glycolytic phenotype, to achieve the effect of anticancer.^[38]^ And also, PTEN has a negative control in the PI3K/Akt/mTOR signaling pathway.^[47]^ PTEN mutations are seen in 30% to 50% of TNBC individuals.^[48]^ As a result, ROS and PTEN mutations can control the amount of HIF-1α by triggering the PI3K/Akt/mTOR signaling pathway.

#### 2.3.3. JAK-STAT signaling pathway.

The development of TNBC is tightly associated with the JAK-STAT signaling pathway. Recent study has shown that the JAK-STAT signaling pathway component STAT3, which is highly expressed in TNBC, is linked to the genesis of tumors, their progression, and the development of treatment resistance.^[49]^ Some scholars had found that STAT3 expression is increased in doxorubicin-resistant TNBC stem cells.^[50]^ According to late research, STAT3 signaling regulates HIF-1 upstream, and when STAT3 is activated, it binds to the HIF-1α promoter and increases HIF-1α transcription.^[39]^ Other scholars found that in MDA-MB-231 cells, IL-6 activated STAT3, and increased HIF-1 expression.^[51]^ Therefore, targeting this pathway can inhibit the expression of HIF-1α.

### 2.4. HIF-1α is controlled by noncoding RNAs

Important regulators of cancer and development are noncoding RNAs.^[52]^ Through controlling HIF-1α, several of these noncoding RNAs can support biological functions associated with tumors, including cell survival, tumor spread, immunological evasion, and metabolic reprogramming.^[53–55]^ In recent years, noncoding RNAs which regulate HIF-1α have been extensively investigated; but their exploration in TNBC is restricted, and some of the mechanisms are yet unclear.

In addition, there are numerous ways whereby lncRNAs control the expression of HIF-1. TNBC cells express MIR210HG more than non-TNBC and nonmalignant MCF10A cells do, and TNBC cells have a specific purpose for it. MIR210HG controls the translational level of HIF-1 expression, raises HIF-1 protein expression levels, and controls the expression of genes involved in glycolysis.^[56]^

To sum up, present research indicates that noncoding RNAs control the expression of HIF-1 in TNBC in a variety of ways. By preserving the stability of the HIF-1 protein and controlling the stability and degree of HIF-1 mRNA translation, they control the expression of the HIF-1 protein.

### 2.5. Epigenetic regulation of HIF-1α

The term “epigenetics” describes a heritable, revocable alteration in gene function that does not involve changing the DNA sequence.^[57]^ It is well known that the classical regulation of epigenetic modification includes DNA methylation and posttranslational histone modification.^[58]^ Epigenetic changes have a close relationship to HIF-1 stability and the regulation of downstream target genes. Moreover, in hypoxic conditions, HIF-1 regulation has a role in both the expression and activity of epigenetic regulators. Among them, TNBC has conducted extensive research on DNA methylation, histone acetylation to HIF-1α, and downstream target gene regulation.

When DNA gets methylated, the methyl group from S-adenosylmethionine is transferred to the cytosine atom at position 5 under the methyltransferase’s catalysis to create 5’-methylcytosine. Gene repression and chromosomal instability are linked to DNA hypermethylation modification, while the opposite is frequently true for DNA hypomethylation.^[59]^ HIF-1α levels and transcriptional activity are changed by DNA methylation. HIF-1 expression and activity were observed to be considerably higher in TNBC samples and MDA-MB-231 cells than in luminal epithelial cells and tissue samples. HIF-1α and glycolytic enzymes (PKM2, lactate dehydrogenase A) hypomethylated DNA in fibroblasts under hypoxic conditions, which encourages the increased glycolytic metabolic activity of TNBC-derived mammary CAF.^[60]^ Because of this, the hypoxia response element in the target gene’s promoter or enhancer undergoes methylation alteration, which alters its binding affinity and consequently its transcriptional activation. In addition, HIF-1 controls DNA demethylase. By transforming 5-methylcytosine into 5-hydroxymethylcytosine, the dioxygenases in the 10 to 11-Translocation (TET) family of enzymes helps DNA become demethylated (5hmC).^[61]^ The TET family of enzymes includes TET1 and TET3, among others. HIF-1 controls TET1 and TET3 expression under hypoxic settings, which in turn coordinately activates the TNF-P38 MAPK signaling pathway to advance TNBC.^[62]^

Histones’ N-terminal tails contain lysine residues that can be acetylated or deacetylated. Histone acetylation enhances transcription by modifying higher-level chromatin structure, reducing links between histones and DNA, and providing binding sites for constitutively active complexes.^[63]^ Histone acetylation is controlled by the enzymes histone acetyltransferase pyruvate dehydrogenase kinase isozyme 1 and histone deacetylase (HDAC). Histone deacetylation caused by HDAC is linked to transcriptional repression, while histone hyperacetylation caused by pyruvate dehydrogenase kinase isozyme 1 is linked to transcriptional activation.^[64]^ The alteration of histone acetylation can control the stability of HIF-1. The acetyltransferase Arrest Defective 1 destabilizes HIF-1 by acetylating the lysine residue (K532) in the ODDD domain, increasing its ubiquitination and proteasomal destruction.^[65]^ It was discovered that HDAC1 deacetylates HIF-1 and inhibits the degradation of its protein in TNBC cell lines. By enlisting the assistance of the HDAC1 to form an MTA/HDAC complex, the MTA1 can improve the stability of HIF-1.^[66]^ Deacetylation of HIF-1 in TNBC keeps it stable and stops future deterioration. The expression level of the HIF-1 protein was decreased by targeted suppression of HDAC. Sphingosine kinase 2, which is found in the nucleus, generates sphingosine-1-phosphate (S1P), which promotes the breakdown of HIF-1 by increasing histone acetylation by blocking HDAC1 and HDAC2.^[67]^ The transcriptional activity of downstream target genes is additionally regulated by histone acetylation. The acetylase activity of P300/CBP modifies the chromatin structure of the target genes, enabling transcription of those genes easier.^[68]^

## 3. HIF-1α is activated in triple-negative BC

HIF-1α controls the transcription of multiple target genes and participates in many important biological processes, including glycolysis, angiogenesis, invasion and metastasis, tumor stem cell enrichment, immune escape, and so on. As a result, HIF-1 alpha promotes tumor growth and proliferation and accelerates the development of TNBC. By identifying HIF-1 target genes, some researchers were able to gauge the quantity of HIF-1 and uncovered 20 unique pathways that are affected.^[69]^

### 3.1. HIF1α pathway is activated by XBP1

Despite adjuvant chemotherapy, TNBC patients have the greatest likelihood of relapse within 1 to 3 years.^[4,70]^ Some researchers administered doxorubicin and XBP1 shRNA to MDA-MB-231 xenograft-bearing mice to study the impact of XBP1 on tumor relapse after chemotherapeutic treatment. Surprisingly, combination therapy not only suppressed or delayed tumor relapse but also stopped tumor development.^[14]^ HIF-1α targets in TNBC are jointly regulated by XBP1 and HIF-1. In the context of a tumor microenvironment, hypoxia enhances XBP1 activation, and active XBP1s interact with HIF-1 to augment and encourage the transactivation of HIF1 target genes that progress cancer.

### 3.2. Both HIF-1α and immune evasion

Tumor immune escape is a phenomenon in which tumor cells use a variety of strategies to avoid being identified and attacked by the host immune system, enabling them to endure and develop.^[71]^ HIF-1α upregulation has importance in tumor immune evasion for hypoxic environments.^[72]^ In TNBC, HIF-1α hinders the tumor-killing activity of immune effector cells by major regulatory cytokines, immune checkpoint molecules, and cell transcription factors. This reduces tumor-killing action while promoting the activation and infiltration of immunosuppressive cells, which then contributes to tumor immune escape.

## 4. Conclusion and future directions

This article offers a fresh approach to the management of TNBC. Because TNBC is controlled by a complicated network, targeting HIF-1 is critical for treatment. There are several therapeutic approaches that target HIF-1α in TNBC, including reducing HIF-1α mRNA levels by focusing on upstream signals, promoting HIF-1α protein degradation and preventing protein synthesis by controlling protein modifications and preventing HIF-1 dimerization from regulating downstream target genes. Several experimental studies have supported these tactics.

Although HIF-1 targeting offers TNBC patients new hope, they nevertheless face difficulties. There are no approved medications for triple-negative BC at the moment. preclinical research is where most of the drugs that control the expression of HIF-1α are concentrated, but actual clinical effects, patient tolerance, and treatment regimens still need to be assessed and confirmed. Furthermore, HIF-1α activation is a complicated process. For instance, radiotherapy and chemotherapy can control the amount of inflammatory mediators released, which in turn can control HIF-1 expression.^[22]^ Combining currently available treatments may be critical for effective treatment of TNBC. In addition, factors such as tumor acidosis, anoxic tumor microenvironment, and increased fluid pressure in the tumor stroma hinder drug delivery and effectiveness.^[73]^ To improve therapeutic effectiveness, more research into appropriate drug carriers and the design of drug delivery systems will be needed. TNBC is also extremely diverse,^[74]^ even if HIF-1α protein is widely expressed in TNBC, it is still important to find reliable biomarkers that can estimate the population’s susceptibility to HIF-1α therapy. Thus, while intensive mechanistic studies are under way, targeting HIF-1 has emerged as a new therapeutic approach for treating BC patients. Novel strategies targeting HIF-1 are likely to be useful in combination with current therapeutic regimens. Future work is warranted to identify more selective HIF-1 inhibitors, to study their mechanism of action, and to incorporate them in clinical trials.

Although HIF-1 targeting offers TNBC patients new hope, they nevertheless face difficulties. There are no approved medications for triple-negative BC at the moment. preclinical studies account for the majority of the work on drugs that control HIF-1 expression; nevertheless, more study is required to determine the drugs real clinical effects, patient tolerance, and dosage regimens. Furthermore, HIF-1 activation is a complicated process. For instance, radiotherapy and chemotherapy can control the amount of inflammatory mediators released, which in turn can control HIF-1 expression.^[22]^ Combination with currently available therapies may be essential for the effective treatment of TNBC. Additionally, variables such tumor acidosis, a hypoxic microenvironment, and increased fluid pressure in the tumor interstitium hinder drug delivery and effectiveness.^[73]^ To improve therapeutic effectiveness, more research into appropriate drug carriers and the design of drug delivery systems will be needed. TNBC is also extremely diverse,^[74]^ Moreover, even if HIF-1 protein is widely expressed in TNBC, it is still important to create reliable biomarkers that can estimate the proportion of the population that will respond favorably to HIF-1 therapy. Finally, targeting HIF-1 will open up new avenues for the therapy of TNBC.

## Author contributions

**Funding acquisition:** Daqing Jiang.

**Methodology:** Jie Bai, Lanxin Hu.

**Resources:** Lihui Liu.

**Supervision:** Lihui Liu, Jie Bai, Daqing Jiang.

**Writing – original draft:** Lihui Liu.

**Writing – review & editing:** Lihui Liu.
